# Tomato (*Lycopersicon esculentum*) Supplementation Induces Changes in Cardiac miRNA Expression, Reduces Oxidative Stress and Left Ventricular Mass, and Improves Diastolic Function

**DOI:** 10.3390/nu7115493

**Published:** 2015-11-19

**Authors:** Bruna L. B. Pereira, Fernanda C. O. Arruda, Patrícia P. Reis, Tainara F. Felix, Priscila P. Santos, Bruna P. Rafacho, Andrea F. Gonçalves, Renan T. Claro, Paula S. Azevedo, Bertha F. Polegato, Katashi Okoshi, Ana A. H. Fernandes, Sergio A. R. Paiva, Leonardo A. M. Zornoff, Marcos F. Minicucci

**Affiliations:** 1 Internal Medicine Department, Botucatu Medical School, Univ Estadual Paulista (UNESP), Botucatu 18618-970, Brazil; bruh_leticia@hotmail.com (B.L.B.P.); fer_nanda893@hotmail.com (F.C.O.A.); priscilaportugal@fait.edu.br (P.P.S.); brunapaola@gmail.com (B.P.R.); andreafgoncalves@gmail.com (A.F.G.); renan_turini@hotmail.com (R.T.C.); paulasa@fmb.unesp.br (P.S.A.); berthafurlan@fmb.unesp.br (B.F.P.); katashi@fmb.unesp.br (K.O.); paiva@fmb.unesp.br (S.A.R.P.); lzornoff@fmb.unesp.br (L.A.M.Z.); 2 Department of Surgery and Orthopedics, Botucatu Medical School, Univ Estadual Paulista (UNESP), Botucatu 18618-970, Brazil; preis@fmb.unesp.br (P.P.R.); felix.tainara@gmail.com (T.F.F.); 3 Chemistry and Biochemistry Department, Institute of Biosciences, Univ Estadual Paulista (UNESP), Botucatu 18618-970, Brazil; angelica@ibb.unesp.br

**Keywords:** tomato, microRNA, oxidative stress, hypertrophy

## Abstract

The aim of this study was to evaluate the effects of tomato supplementation on the normal rat heart and the role of oxidative stress in this scenario. Male Wistar rats were assigned to two groups: a control group (C; *n* = 16), in which animals received a control diet + 0.5 mL of corn oil/kg body weight/day, and a tomato group (T; *n* = 16), in which animals received a control diet supplemented with tomato +0.5 mL of corn oil/kg body weight/day. After three months, morphological, functional, and biochemical analyses were performed. Animals supplemented with tomato had a smaller left atrium diameter and myocyte cross-sectional area (CSA) compared to the control group (C group: 474 (415–539); T group: 273 (258–297) µm^2^; *p* = 0.004). Diastolic function was improved in rats supplemented with tomato. In addition, lipid hydroperoxide was lower (C group: 267 ± 46.7; T group: 219 ± 23.0 nmol/g; *p* = 0.039) in the myocardium of rats supplemented with tomato. Tomato intake was also associated with up-regulation of miR-107 and miR-486 and down-regulation of miR-350 and miR-872. In conclusion, tomato supplementation induces changes in miRNA expression and reduces oxidative stress. In addition, these alterations may be responsible for CSA reduction and diastolic function improvement.

## 1. Introduction

Cardiovascular disease (CVD) is the major cause of mortality in the United States (US). According to the World Health Organization, in 2030, CVD will be responsible for almost 23.6 million deaths worldwide [1]. A diet rich in fruits and vegetables is associated with a reduced cardiovascular risk [2]. Modulation of oxidative stress, inflammation, immune response, endothelial function, blood pressure, and lipid metabolism are mechanisms that could explain the beneficial effects of fruit and vegetable consumption [2]. Thus, currently, dietary modification and food supplements are attractive methods for CVD prevention and management. Among these interventions, consumption of tomato and tomato-based foods is associated not only with a lower risk of cancer, such as prostate cancer, but also with a decrease in cardiovascular risk [3,4].

Tomato fruits (*Lycopersicon esculentum*) provide a number of essential nutrients and other bioactive components to the diet. Annual fresh tomato consumption is 18 kg per capita in Europe and 8 kg per capita in the US [5]. Among the many tomato components, lycopene is the most investigated. Lycopene accounts for almost 90% of total carotenoids in tomatoes [6]. However, tomatoes also contain β-carotene, folate, phenolic compounds, and vitamins C and E [5]. Lycopene is a fat-soluble pigment that gives tomatoes their red color. This hydrocarbon carotenoid has the strongest singlet oxygen-quenching activity of all carotenoids [7,8]. In addition, lycopene regulates the activity of E2-related factor 2 (Nrf-2), a transcription factor that stimulates the transcription of antioxidant enzymes, such as superoxide dismutase (SOD), catalase (CAT), and thioredoxin [7,8].

Several clinical trials have evaluated lycopene supplementation and tomato-based food interventions on traditional and emerging CVD risk factors [9,10,11]. In healthy individuals or in patients with cardiovascular risk factors, lycopene supplementation reduced oxidative damage and blood pressure and improved the lipid profile [2,9,10,11]. Experimental studies have also shown that in aggressive heart models, such as myocardial infarction, doxorubicin cardiotoxicity, and obesity, lycopene and tomato supplementation attenuated cardiac dysfunction, inflammation, and oxidative stress [12,13,14,15]. However, the direct effect of tomato supplementation and the regulation of microRNA expression in the myocardium of normal rats have not yet been evaluated.

MicroRNAs are small, non-coding RNA molecules of approximately 22 nucleotides that act as post-transcriptional regulators of gene expression. Currently, miRNAs are considered key regulators that are involved in several cardiovascular diseases, such as left ventricular hypertrophy, heart failure, hypertension, and ischemic heart diseases [16]. The study of miRNAs could help in the understanding of the protective role of tomato and tomato based-foods on cardiovascular mortality.

Thus, the aim of this study was to evaluate the effects of tomato supplementation on the normal rat heart and the role of oxidative stress in this scenario.

## 2. Experimental Section

All of the experiments and procedures were approved by the Animal Ethics Committee of Botucatu Medical School and were performed in accordance with the National Institute of Health’s Guide for the Care and Use of Laboratory Animals.

Male Wistar rats weighing 200–250 g were assigned to two groups: a control group (C; *n* = 16), in which animals received a control diet + 0.5 mL of corn oil/kg body weight/day, and a tomato group (T; *n* = 16), in which animals received a control diet supplemented with tomato + 0.5 mL of corn oil/kg body weight/day. Tomato supplementation in the diet was equivalent to 1 mg of lycopene/kg body weight/day [17,18]. Water was supplied *ad libitum*. The dietary intake was recorded daily. The rats were observed for three months, after which morphological, functional, and biochemical analyses were performed.

### 2.1. Diet Preparation

Fresh tomatoes were cooked for 5 min at 80 °C and placed in tap water. The tomato skin was removed manually. Then, they were triturated and dried at 65 °C for 48 h. The lycopene content of the tomato powder was analyzed by high-performance liquid chromatography (HPLC) according to the method described by Riso and Yeum [19,20]. The lycopene concentration was 5.9 μg/mg of tomato powder. For each kg of control chow that was added, 4.2 g of tomato powder was used to supply the equivalent of 1 mg of lycopene/kg body weight/day. This amount is equivalent to 700 mg of tomato/kg/day in humans, or approximately half of tomato/day in an adult who weighs 60 kg [21].

### 2.2. Echocardiographic Analysis

After three months, all animals were weighed and evaluated by a transthoracic echocardiographic exam (General Electric Medical Systems, Vivid S6, Tirat Carmel, Israel). All of the measurements were taken by the same observer, according to the method recommended by the American Society of Echocardiography [22]. The following structural variables were measured: left atrium (LA) diameter, left ventricle (LV) diastolic and systolic dimensions (LVDD and LVSD, respectively), and LV diastolic posterior wall thickness (PWT). The relative wall thickness (RWT) was determined as (2 × PWT)/LVDD. The velocities of transmitral diastolic flow (E and A velocities) were obtained from the apical four-chamber view. The E/A ratio, the isovolumetric relaxation time, and the isovolumetric relaxation time corrected by the heart rate (IRT/RR^0.5^) were used as indices of LV diastolic function. Systolic function was assessed based on the endocardial fractional shortening (FS) and posterior wall shortening velocity (PWSV).

### 2.3. Morphometric Analysis

After the echocardiographic analyses, the rats were euthanized and the right and left ventricles (including the interventricular septum) were dissected, separated, and weighed. Transverse sections of the LV were fixed in 10% buffered formalin and paraffin embedded. Five-micron-thick sections were stained with hematoxylin and eosin (HE). The myocyte cross-sectional area (CSA) was determined for a minimum of 50 myocytes per HE-stained cross section. The measurements were obtained from digital images that were collected with a video camera attached to a Leica microscope; the images were analyzed with the Image-Pro Plus 3.0 software program (Media Cybernetics; Silver Spring, MD, USA). The CSA area was measured with a digital pad, and the selected cells were transversely cut so that the nucleus was in the center of the myocyte [23].

### 2.4. Myocardial Lipid Hydroperoxide and Antioxidant Enzyme Analysis

Left ventricle samples (200 mg) were homogenized in 5 mL of 0.1 M cold sodium phosphate buffer, pH 7.4, containing 1 mM ethylenediaminetetraacetic acid (EDTA). Tissue homogenates were prepared with a motor-driven Teflon glass Potter Elvehjem tissue homogenizer (for 1 min at 100 rpm) immersed in ice water. The homogenate was centrifuged at 10,000 rpm for 15 min, and the supernatant was used to determine the total protein concentration as described previously. Lipid hydroperoxide was measured through hydroperoxide-mediated oxidation of Fe^2+^, as previously published [24]. Glutathione peroxidase (GSH-Px), superoxide dismutase (SOD), and catalase (CAT) were assessed as previously specified [24,25,26]. Enzyme activities were determined at 25 °C using a micro-plate reader (lQuant-MQX 200 with Kjunior software connected to computer system control, BioTec Instruments, Winooski, VT, USA). The spectrophotometric determinations were performed in a Pharmacia Biotech spectrophotometer with a temperature-controlled cuvette chamber (UV/visible Ultrospec 5000 with Swift II Applications software connected to a computer system control, 974213, Cambridge, UK). All reagents were from Sigma (St. Louis, MO, USA) [24].

### 2.5. Nrf-2, Type I and III Collagen Analysis

Samples of LV were added to Tris-Triton buffer (10 mM Tris (pH 7.4), 100 mM NaCl, 1 mM EDTA, 1 mM ethylene glycol tetraacetic acid (EGTA), 1% Triton X-100, 10% glycerol, 0.1% Sodium dodecyl sulfate (SDS), 0.5% deoxycholate, 1 nM EDTA, 1 mM EGTA and a mixture of protease inhibitors (1 mM sodium orthovanadate, 1 mM sodium fluoride and 1% leupeptin, aprotinin and pepstatin)) to detect type I (rabbit polyclonal IgG, sc8784R, Santa Cruz Biotechnology, Inc., Dallas, TX, USA) and III (mouse monoclonal IgG1, ab6310, Abcam, Inc., Cambridge, UK) collagen. Nuclear protein extraction from the LV was performed with the NE-PER Nuclear Extraction Reagents kit (Pierce Biotechnology, Rockford, IL, USA). Nuclear extracts were used to detect Nrf-2 (C-20, rabbit polyclonal IgG, sc722, Santa Cruz Biotechnology, Inc.). Secondary antibodies were used according to the manufacturer's recommendations. GAPDH (GAPDH (6C5), mouse monoclonal IgG1, sc32233, Santa Cruz Biotechnology, Inc.) was used for normalization [27].

### 2.6. Left Ventricular miRNA Expression

Six animals in each group were used for miRNA expression analyses. RNA was extracted from LV samples with the Recover All Total Nucleic Acid Isolation Kit (Ambion/Life Technologies, Carlsbad, CA, USA). The RNA quality and concentration were determined with a NanoDrop 8000 (Thermo Scientific, Waltham, MA, USA). Global miRNA expression analysis was determined using TaqMan Array Rodent MicroRNA Cards (A + B card sets v3.0; Life Technologies), according to the manufacturer’s instructions. Data analysis was performed using global normalization in RQ Manager v1.2 software (Life Technologies) [28]. This method ensures that the most stable set of endogenous control miRNAs are used for data normalization. miRNA data has been generated following the MIQE guidelines [29]. In order to visualize whether expression of significantly deregulated miRNAs (fold change > 2 for up- or down-regulation) was associated with sample clustering, data were subjected to hierarchical clustering analysis using Eisen Cluster [30]. In this analysis, unsupervised average linkage hierarchical clustering was applied to organize miRNAs and samples into groups. Cluster of samples (dendogram) and cluster of miRNA expression was visualized by Tree View [30].

### 2.7. Statistical Analysis

Data are expressed as the mean ± SD or as the median (lower quartile-upper quartile). Comparisons between groups were performed using Student’s *t*-test when data were normally distributed and the Mann–Whitney test when the distributions were non-normal. Data analysis was performed with SigmaStat for Windows v2.03 (SPSS Inc., Chicago, IL, USA). The significance level was 5%.

## 3. Results

There was no difference in the mean daily dietary intake between the groups (C group: 21.6 (21.2–22.1) g; T group: 22.9 (21.2–23.3) g; *p* = 0.105). In addition, body weight (BW) was not different between the groups. Morphological and functional echocardiographic data are presented in Table 1. Animals supplemented with tomato had a lower LA and LA corrected by BW. There were no differences in other morphological variables. Variables that evaluate systolic function were also not different between the groups. However, IRT/RR^0.5^ was lower in the tomato group; the lower LA and IRT/RR^0.5^ suggest an improvement in diastolic function in rats supplemented with tomato.

**Figure 1:**
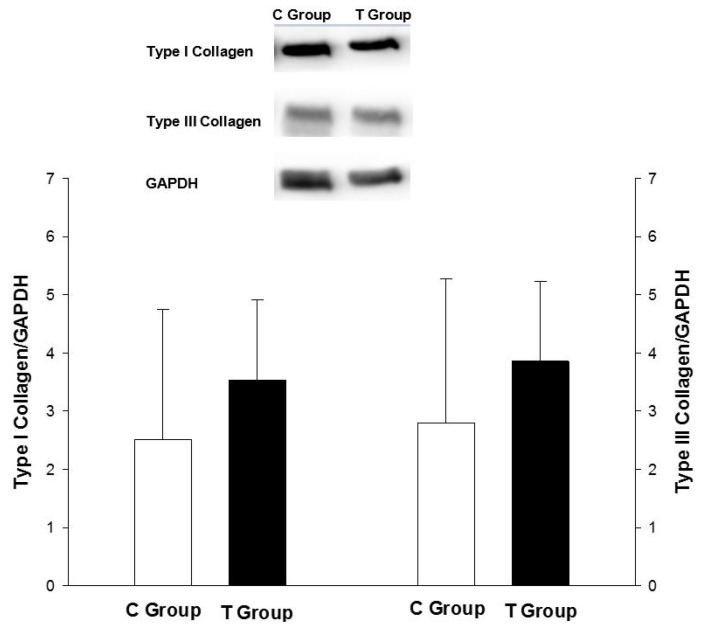
Left ventricle collagen type I and III expression.

Morphological data are listed in Table 2. The tomato group had a lower right ventricular weight and RV/BW compared to the control group. There was no difference in the LV weight between the groups. There was also no difference in LV expression of type I and type III collagen (Figure 1). Regarding the CSA, the tomato group had lower values compared to the control group (C group: 474 (415–539) µm^2^ (*n* = 6); T group: 273 (258–297) (*n* = 5) µm^2^; *p* = 0.004).

**Table 1:** Morphological and functional data evaluated by echocardiography.

Variable	Control Group (*n* = 16)	Tomato Group (*n* = 16)	*p* Value
BW (g)	434 (419–444)	456 (422–486)	0.090
LVDD (mm)	7.87 ± 0.43	7.72 ± 0.38	0.305
LA (mm)	5.31 ± 0.30	5.05 ± 0.33	0.029
LVDD/BW (mm/kg)	18.2 ± 1.27	17.3 ± 1.9	0.154
LA/BW (mm/kg)	12.3 ± 0.84	11.3 ± 1.19	0.016
PWT (mm)	1.31 ± 0.07	1.31 ± 0.07	0.879
RWT	0.34 ± 0.02	0.34 ± 0.02	0.375
HR (bpm)	259 ± 25.0	265 ± 26.2	0.454
FS (%)	50.0 ± 4.71	47.9 ± 3.32	0.148
PWSV (mm/s)	36.9 ± 4.21	37.5 ± 4.48	0.730
E wave (ms)	77.1 ± 8.60	73.9 ± 5.00	0.219
A wave (ms)	48.4 ± 4.86	44.3 ± 6.72	0.071
E/A	1.61 ± 0.19	1.70 ± 0.25	0.243
EDT (ms)	46.9 ± 8.29	45.7 ± 8.13	0.685
IRT/RR^0.5^ (ms)	54.2 ± 5.91	48.8 ± 7.52	0.030

BW: body weight; HR: heart rate; LVDD: LV end-diastolic dimension; LA: left atrium; PWT: LV posterior wall thickness; RWT: relative wall thickness; HR: heart rate; FS: endocardial fractional shortening; PWSV: posterior wall shortening velocity; E/A: peak velocity of early ventricular filling/peak velocity of transmitral flow during atrial contraction; EDT: E wave deceleration time; IRT/RR^0.5^: isovolumetric relaxation time adjusted by heart rate. Data are expressed as the mean ± SD or as the median (lower quartile–upper quartile).

**Table 2:** Morphometrical analysis.

Variable	Control Group (*n* = 15)	Tomato Group (*n* = 8)	*p* Value
RV (g)	0.25 (0.21–0.31)	0.17 (0.14–0.18)	<0.001
RV/BW	0.06 (0.05–0.07)	0.04 (0.03–0.04)	<0.001
LV (g)	0.96 (0.81–1.08)	0.90 (0.84–0.96)	0.540
LV/BW	0.22 (0.19–0.25)	0.20 (0.18–0.20)	0.129

RV: right ventricle; BW: body weight; LV: left ventricle. Data are expressed as the mean ± SD or as the median (lower quartile–upper quartile).

**Figure 2:**
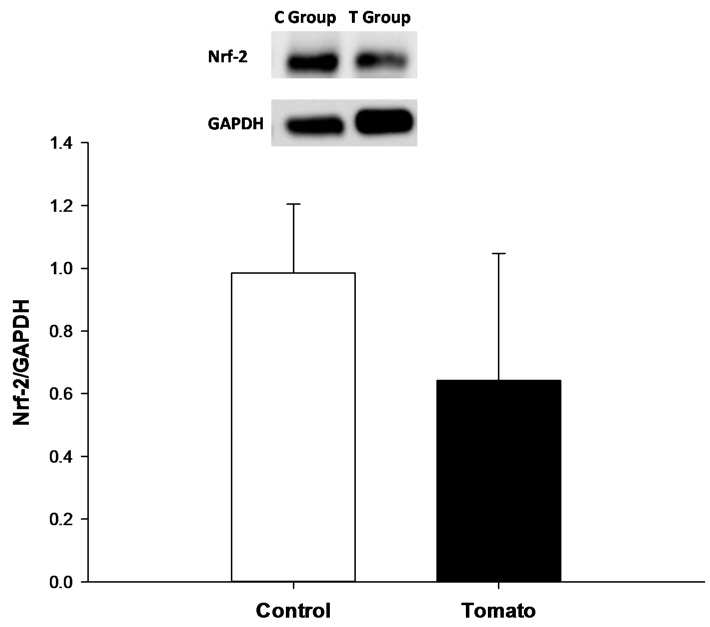
Left ventricle Nrf-2 expression.

The oxidative stress marker lipid hydroperoxide was also lower in the myocardium of rats supplemented with tomato (Table 3). We observed higher CAT and GSH-Px activity and lower SOD activity in the tomato group compared to the control group. However, cardiac expression of nuclear Nrf-2 was similar between the groups (Figure 2).

**Table 3:** Left ventricle oxidative stress.

Variable	Control Group (*n* = 6)	Tomato Group (*n* = 8)	*p* Value
LH (nmol/g)	267 ± 46.7	219 ± 23.0	0.039
CAT (µmol/g)	72.8 ± 7.14	95.3 ± 18.3	0.015
SOD (nmol/g)	16.2 ± 2.19	12.8 ± 1.10	0.002
GSH-Px (nmol/g)	38.5 ± 6.25	91.3 ± 6.36	<0.001

LH: lipid hydroperoxide; CAT: catalase; SOD: superoxide dismutase; GSH-Px: glutathione peroxidase. Data are expressed as the mean ± SD.

**Figure 3:**
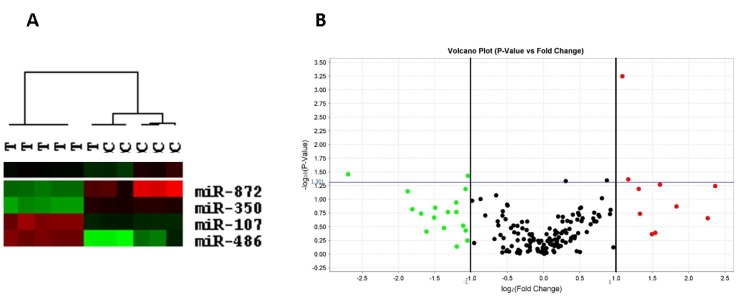
(**A**) Dendogram obtained by hierarchical clustering of 11 samples, six from experimental and five from control groups. Tree view shows data visualization representing miRNAs and samples. miRNA cluster shows over-expressed (red) and under-expressed (green) miRNAs. Black color means unchanged; and (**B**) volcano plot of differentially expressed miRNAs in tomato *versus* control group.

Clustering results showed that samples from the tomato group clustered separately from control samples. Regarding LV miRNA expression, tomato intake was significantly associated with the up-regulation of miR-107 (*p* = 0.043) and miR-486 (*p* = 0.001) and the down-regulation of miR-350 (*p* = 0.035) and miR-872 (*p* = 0.037) (Figure 3).

## 4. Discussion

The objective of this study was to evaluate the effects of tomato supplementation on the normal rat heart and the role of oxidative stress in this scenario. Our data showed that tomato induced changes in miRNA expression and reduced oxidative stress. In addition, these alterations could be responsible for the CSA reduction and the diastolic function improvement.

The reason for an improvement in cardiac health resulting from tomato consumption is not yet understood. Although tomato supplementation influences oxidative stress, inflammation, blood pressure, and the lipid profile, the direct effects of tomato in the myocardium had not yet been studied [9,10,11]. It was important to observe such effects in our study; animals supplemented with tomato had a lower CSA. Left ventricular mass and hypertrophy are associated with an increased risk of sudden death and all-cause mortality [31,32]. Although left ventricular weight was lower in the tomato group, the difference was not statistically significant. However, the myocyte CSA is another important marker for evaluating cardiac hypertrophy. Thus, a reduction in the left ventricular mass could be one of the preventative effects of tomato supplementation in the heart.

Left ventricular mass is also associated with diastolic function. In our study, the smaller CSA values in the tomato group were accompanied by smaller LA diameters and IRT/RR^0.5^. In addition, worse diastolic function leads to an increased LA diameter, increased pulmonary pressure, and RV hypertrophy. Our rats supplemented with tomato also had lower RV weight compared to the control group. Therefore, these alterations suggest that tomato supplementation improves diastolic function in healthy animals, which is associated with a decreased RV mass.

The major effects of tomato supplementation are related to oxidative stress. Oxidative stress occurs when there is an imbalance between reactive oxygen species (ROS) production and antioxidant systems. It is well accepted that oxidative stress induces several deleterious cardiac alterations including mitochondrial dysfunction, lipid peroxidation, DNA damage, calcium handling modifications, cellular dysfunction, metalloproteinases activation, fibrosis, hypertrophy, and cell death. However, the importance of ROS is not restricted to oxidative damage; at nanomolar concentrations, they play an important role in transcription factor modulation and in signal transduction pathways [33,34]. Lycopene in tomatoes has the strongest singlet oxygen-quenching activity of all carotenoids. In addition, it regulates Nrf-2 activity and modulates the transcription of antioxidant enzymes [7,8]. In our study, tomato supplementation decreased lipid hydroperoxide, a biomarker of lipoperoxidation. In addition, the tomato group had higher CAT and GSH-Px activity, suggesting an improvement in antioxidant defenses. However, left ventricular expression of Nrf-2 was not different in the group with tomato supplementation. Our study only evaluated the animals after three months of tomato supplementation; therefore, we cannot exclude that Nrf-2 was overexpressed during the experimental protocol. Thus, the antioxidant effect could be another mechanism that explains the cardiac protection with tomato supplementation.

All of these alterations that were observed with tomato supplementation could be, at least in part, due to changes in cardiac miRNA expression. Recent evidence suggests that miRNAs play an important role in the progression of heart failure by targeting genes that govern diverse functions in cardiac remodeling processes, such as myocyte hypertrophy, oxidative stress, and myocardial fibrosis [35]. To the best of our knowledge, this is the first study to investigate the association of tomato supplementation and miRNA expression in the LV. Few studies have evaluated miRNA expression after tomato or lycopene supplementation. Ahn *et al.* showed that lycopene inhibits hepatic steatosis by normalizing miR-21 expression, which was down-regulated in hepatic steatosis [36]. In our study, tomato up-regulated the LV expression of miR-107 and miR-486, and down-regulated the expression of miR-350 and miR-872.

To date, several miRNAs that modulate key components of myocyte hypertrophy pathways have been identified. Specifically, miRNAs miR-1, 21, 23, 133, and 350 play important roles in this scenario [35]. Ge *et al.* showed that miR-350 was up-regulated in rat hearts in response to late-stage transverse aortic constriction and that miR-350 is a critical regulator of pathological cardiac hypertrophy and apoptosis in rats [37]. Thus, the down-regulation of miR-350 observed in the heart of rats supplemented with tomato could be responsible for the decrease in the CSA that we showed in this study. Moreover, miR-486 in skeletal muscle was linked to the mTOR pathway as a key pathway in muscle mass regulation [38]. Its function in the myocardium has not yet been demonstrated.

miR-1, 133, and 872 also precipitate changes in cardiac oxidative stress. Interestingly, miR-872 contributes to the production of proinflammatory adipocytokines, oxidative damage, and apoptosis by inhibiting heme oxygenase-1 [39]. Thus, the down-regulation of miR-872 could explain the decrease in oxidative stress in the tomato group.

Meng *et al.* showed that miR-107 was up-regulated in hypoxia and prevented endothelial progenitor cell differentiation via down-regulation of the transcription factor hypoxia-inducible factor (HIF)-1β [40]. HIF-1α and HIF-1β form heterodimers and modulate the transcription of genes associated with angiogenesis, inflammation, cell proliferation, and glucose metabolism. The physiological mechanism of miR-107 in the heart has not yet been studied; however, by decreasing HIF expression, it could be used as an anti-ischemic agent [40]. Thus, the overexpression of miR-107 could represent another potential molecular mechanism to explain our results.

## 5. Conclusions

In conclusion, tomato supplementation induces changes in miRNA expression and reduces oxidative stress. In addition, these alterations could be responsible for the CSA reduction and diastolic function improvement. Further studies should be performed to investigate the potential targets of tomato and lycopene in cardiovascular disease prevention and management.
